# HDR brachytherapy in vivo source position verification using a 2D diode array: A Monte Carlo study

**DOI:** 10.1002/acm2.12360

**Published:** 2018-06-01

**Authors:** Joel Poder, Dean Cutajar, Susanna Guatelli, Marco Petasecca, Andrew Howie, Joseph Bucci, Anatoly Rosenfeld

**Affiliations:** ^1^ Centre of Medical Radiation Physics University of Wollongong Wollongong NSW Australia; ^2^ St George Hospital Cancer Care Centre Kogarah NSW Australia

**Keywords:** brachytherapy, diode, *in vivo*, Magic Plate, source tracking

## Abstract

**Purpose:**

This study aims to assess the accuracy of source position verification during high‐dose rate (HDR) prostate brachytherapy using a novel, in‐house developed two‐dimensional (2D) diode array (the Magic Plate), embedded exactly below the patient within a carbon fiber couch. The effect of tissue inhomogeneities on source localization accuracy is examined.

**Method:**

Monte Carlo (MC) simulations of 12 source positions from a HDR prostate brachytherapy treatment were performed using the Geant4 toolkit. An Ir‐192 Flexisource (Isodose Control, Veenendaal, the Netherlands) was simulated inside a voxelized patient geometry, and the dose deposited in each detector of the Magic Plate evaluated. The dose deposited in each detector was then used to localize the source position using a proprietary reconstruction algorithm.

**Results:**

The accuracy of source position verification using the Magic Plate embedded in the patient couch was found to be affected by the tissue inhomogeneities within the patient, with an average difference of 2.1 ± 0.8 mm (*k* = 1) between the Magic Plate predicted and known source positions. Recalculation of the simulations with all voxels assigned a density of water improved this verification accuracy to within 1 mm.

**Conclusion:**

Source position verification using the Magic Plate during a HDR prostate brachytherapy treatment was examined using MC simulations. In a homogenous geometry (water), the Magic Plate was able to localize the source to within 1 mm, however, the verification accuracy was negatively affected by inhomogeneities; this can be corrected for by using density information obtained from CT, making the proposed tool attractive for use as a real‐time *in vivo* quality assurance (QA) device in HDR brachytherapy for prostate cancer.

## 1 INTRODUCTION

When used in combination with external beam radiotherapy (EBRT) in the form of a boost, high‐dose rate (HDR) brachytherapy has been shown to be a safe and effective treatment modality for intermediate‐ and high‐risk prostate cancer.^1^, ^2^ Despite recent technological developments in the field of brachytherapy, such as image‐guided brachytherapy,^3^, ^4^ treatment planning,^5^ electromagnetic tracking,^6^ and applicator development,^7^, ^8^, ^9^ poor execution of HDR prostate brachytherapy can still have a significant effect on patient outcomes. Furthermore, the incidences of errors that may occur in the practice of HDR prostate brachytherapy is relatively unknown, and limited options exist for independent routine monitoring of treatment delivery. There are a number of published documents by the International Commission on Radiological Protection (ICRP)^10^, ^11^ as well as the International Atomic Energy Association (IAEA)^12^ describing errors that have occurred in HDR brachytherapy. Many of these errors are related to human miscalculations, and less often, due to machine or computational malfunction. The likelihood of remote afterloader malfunction is generally considered extremely low; however, small deviations from the plan in source dwell position and time can result in significant errors in the dose delivered to the patient.^13^

The American Association of Physicists in Medicine (AAPM) Radiation Therapy Task Group (TG) No. 59^14^ recommends that institutions employ a quality assurance (QA) program that exploits redundancy and review the entire treatment planning and delivery process to isolate any actions susceptible to errors. The report suggests that incidence of these errors may be reduced by the introduction of pretreatment QA in the time between treatment planning and delivery. Further to this, another AAPM Report from TG 56^15^ recommends that the source position, source dwell time, and transit time be quantified by the medical physicist on a regular basis. A combination of these regular and pretreatment QA checks, along with a well‐documented treatment planning and delivery protocol will go a long way to ensuring safe and successful delivery of HDR brachytherapy treatment plans. However, this type of QA program will not safeguard the HDR brachytherapy delivery from all types of errors. An ideal system for HDR brachytherapy treatment verification should be able to provide real‐time identification of the dwell positions, measure the dwell and transit times, and compare these parameters with the prescribed treatment plan both before and during treatment.^16^

Real‐time source identification of dwell positions during HDR prostate brachytherapy treatments based on electronic portal imaging devices (EPIDs) have been performed previously.^17^, ^18^, ^19^ In one study, the authors retrospectively compared the planned vs measured source positions using an EPID embedded into the couch for eight treatment fractions, and the mean linear distance between the planned and measured dwell positions was found to be 1.8 mm (range 0.7–3.9 mm).^17^ However, these EPID‐based devices suffer from low frame rates and slow readout electronics, resulting in loss of data for short dwell times.^18^

This study aims to investigate the feasibility of using a two‐dimensional (2D) diode array, the “Magic Plate” (MP), developed at the Centre for Medical Radiation Physics (CMRP), University of Wollongong (UoW), Australia, for in vivo source position verification of a HDR prostate brachytherapy treatment. Previous studies performed with the MP in homogeneous phantom media and have reported source localization accuracy of less than 1 mm^16^, ^20^, ^21^ and temporal resolution of 1 ms,^20^ making it an ideal device for real‐time source position verification. It is hypothesized that the introduction of heterogeneous media associated with the patient geometry may compromise the accuracy of source localization using this device.

To assess the feasibility of source localization using the MP in the presence of patient‐related heterogeneities, MC simulations were performed using the Geant4 toolkit (v4.10.01).^22^, ^23^ During the HDR prostate brachytherapy treatment simulations, the Flexisource Ir‐192 source (Isodose Control, Veenendaal, the Netherlands) was simulated inside a voxelized patient geometry, and the dose deposited within the sensitive volume of each detector in the couch embedded 11 × 11 diode array was evaluated. The simulated detector dose was then used to determine the distance of all detectors in the array to each of the simulated source positions. Finally, the source position was determined using an iterative procedure where the source position is first estimated, and then repeatedly refined based upon the agreement of the predicted geometric distance from the source to the detectors against those determined by the detectors in the array.

## 2 MATERIALS AND METHODS

### 2.A Ir‐192 Flexisource

**Figure 1:**
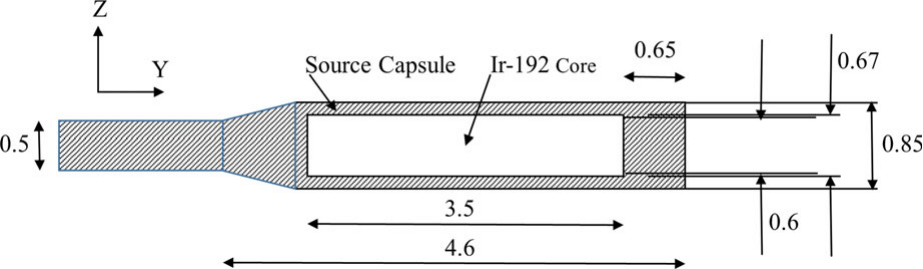
Schematic of Flexisource Ir‐192 source^24^ modeled in this study. All dimensions are in millimeters.

The geometric design of the Flexisource model was obtained from the study performed by Granero et al.^24^ and is shown in Fig. 1. A detailed description of the Flexisource model used in the simulations is included in Appendix I.

### 2.B Magic Plate diode array

The MP is a 2D silicon diode array developed at the CMRP, UoW, Australia, originally as a tool for intensity modulated radiation therapy (IMRT) QA.^25^, ^26^ The MP has since been validated as a tool for Ir‐192 source position verification with focus on pretreatment quality assurance.^16^, ^21^

**Figure 2:**
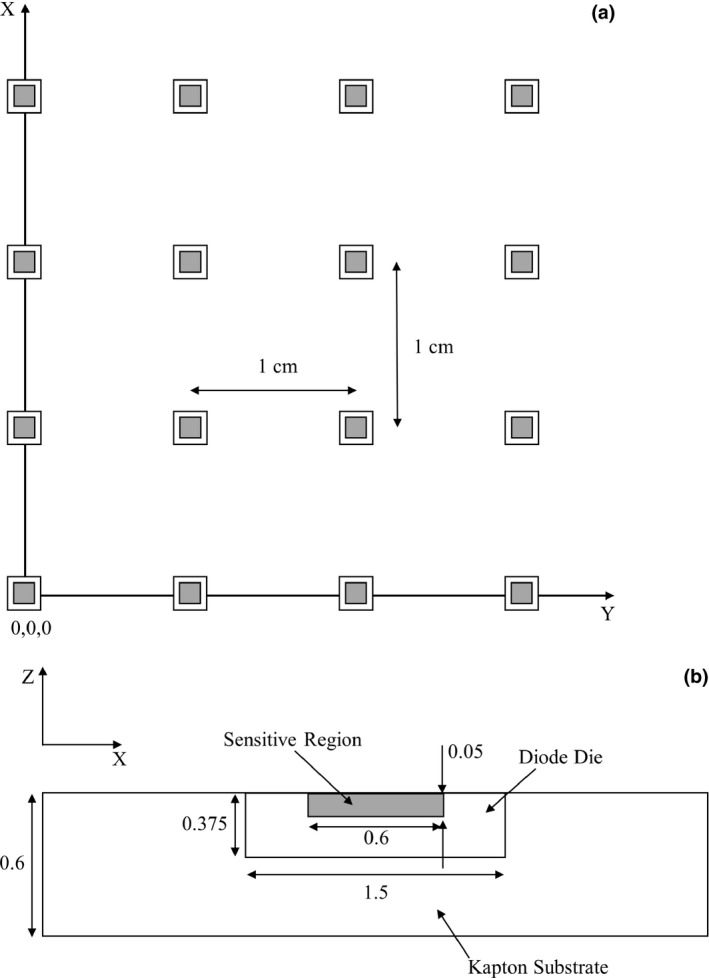
(a) Schematic of the Magic Plate diode spacing, the origin of the coordinate system is defined as the bottom left corner diode of the Magic Plate, (b) Close up of the diode design (distances in mm).

The MP consists of an 11 × 11 array of epitaxial diodes mounted on a 0.6 mm Kapton substrate using the “drop‐in” technique. The structure of the MP is modeled in the Geant4 MC simulations in this study using the description by Wong, et al.^25^ and is shown in Figs. 2(a) and 2(b).

### 2.C TG‐43 simulations

Before using the Flexisource model in MC studies, the physics modeling within the prospective MC simulations was validated against benchmark data via TG‐43 simulations. The AAPM TG43‐U1 report^27^ recommends that MC simulations used to obtain TG‐43 parameters be performed with the source placed in the center of a spherical 400 mm radius water phantom, so as to simulate an unbounded phantom.^28^ The phantom geometry used in this simulation was a 400 mm radius spherical phantom comprised of liquid water with physical density 0.998 g/cm^3^. The density of 0.998 g/cm^3^ was chosen so as to simulate the density of liquid water at 22°C as is recommended in TG43‐U1.^27^

The spectrum of gamma energies emitted from the Ir‐192 source was obtained from the NuDat database.^29^ The *β* spectrum was not considered in the study since its contribution to the dose delivered beyond the stainless steel shell is negligible.^24^, ^30^ A total of 10^9^ primary photons were generated for each simulation run, and a total of 10 simulation runs were performed. Results from each run were averaged and the standard deviation (k = 1) calculated.

The interaction processes for photons (the photoelectric effect, Compton scattering and Rayleigh scattering) are modeled using the Geant4 Livermore Low Energy Package. The interactions cross‐sections tabulation was taken from the EPDL97 database.^31^ In order to improve the efficiency of the simulations, the linear track‐length kerma estimator^32^ was utilized with a photon cutoff energy of 250 eV.

Interactions for electrons (multiple scattering, ionization, and bremsstrahlung) are also modeled using the Geant4 Livermore Low Energy Package. Secondary particles with range less than 1 μm are assumed to deposit the dose locally in the interaction voxel.^30^

To obtain the dose rate in polar coordinates and calculate the TG‐43 parameters, the dose was scored in spherical sections with thickness of 0.5 mm (from 0 to 200 mm) and angular resolution of 1° (from 0° to 180°) concentric to the longitudinal axis of the source. The thickness and resolution of the voxels were chosen so as to ensure the effects of volume averaging was less than 0.1% for distances greater than 5 mm from the source.^33^ To calculate the absorbed dose in each of the spherical sections, the total energy deposited in each section was obtained and divided by the total mass of the section.

The Ir‐192 source is located at the origin of the calculation volume with its longitudinal axis placed along the y axis of the coordinate system, as shown in Fig. 1. To calculate the radial dose function, the absorbed dose along the z axis was normalized to the absorbed dose at 10 mm from the center of the source, before being divided by the normalized (at z = 10 mm) line source geometry function, as per eq. (A6) of the AAPM TG43‐U1 report.^27^

To calculate the 2D anisotropy function, for a given radial distance from the center of the source (r) the absorbed dose was plotted as a function of the angle from the longitudinal axis (θ), normalized to θ* *= 90°, and then divided by the normalized (again at θ* *= 90°) line source geometry function, as shown in Equation 8 of the AAPM TG43‐U1 report.^27^

### 2.D Source position verification simulations

Once the Flexisource model had been validated through TG‐43 simulations, the same source was simulated inside a voxelized patient model, and the dose deposited in each of the diodes in the carbon couch embedded MP was tallied. The MP was modeled in the source position verification simulations as described in Section 2.B, embedded inside a 120‐mm thick carbon couch, offset 5 mm from its anterior surface.

**Figure 3:**
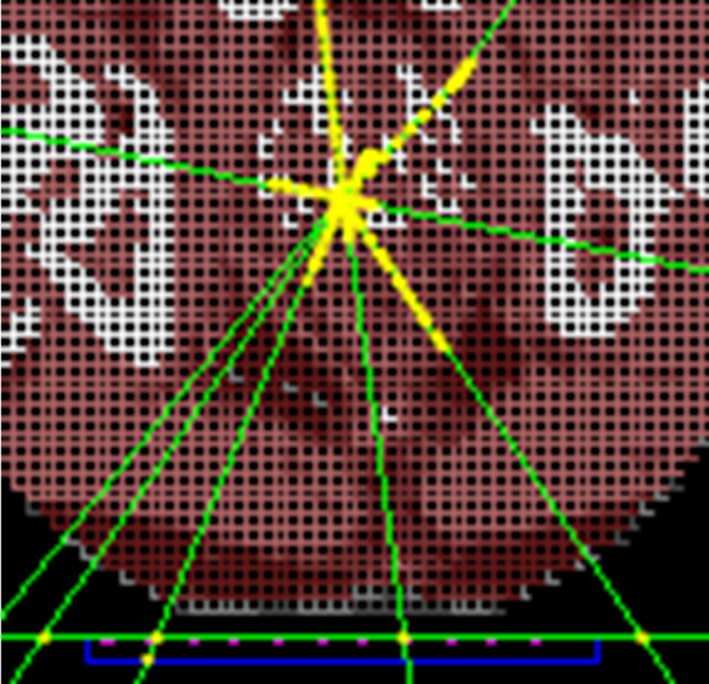
Partial axial view of voxelized patient geometry in Geant4 source position simulations. The carbon couch is shown below the patient geometry outlined in green, the Kapton substrate in blue, and the diode array in pink.

The patient model was created by converting a DICOM CT study set from a prostate HDR brachytherapy treatment into a voxelized model that can be used in Geant4, as shown in Fig. 3. This was achieved by first converting the Hounsfield unit (HU) numbers to a mass density value using a CT–density curve, and then converting from mass density to a material using a look up table.^34^, ^35^, ^36^ Once imported into the simulation, a geometrical phantom is created, within which is an array of voxels containing the materials (and their compositions) determined from the HU numbers.^36^ The compositions and the densities of materials used in the simulations were obtained from the AAPM TG 186 Report.^37^ The voxel size was set to 3 × 3 × 3 mm^3^ in this study, to model an accurate geometrical definition of patient‐related inhomogeneities and prevent prohibitively long simulation times.

A selection of 12 source positions from a HDR prostate brachytherapy treatment plan created in the Oncentra Brachytherapy treatment planning system (Elekta Brachytherapy, Veenendaal, the Netherlands) were used in the simulations. The source position coordinates were selected as three consecutive source positions from four catheters. The catheters were selected such that they spanned the extent of the prostate (L–R and A–P), to determine if consecutive source positions along a catheter could be localized by the MP at the maximum and minimum distances expected in a clinical HDR prostate brachytherapy treatment. The step size of the source used in the treatment planning system was 3 mm.

The same geometrical source model as described in Section 2.A was used in the simulations, along with the same gamma spectrum and interaction processes described in Section 2.C. To prevent overlapping volumes, which causes tracking errors in simulations, a parallel geometry was used to place the source within the patient geometrical phantom at the planned dwell positions.^38^ To calculate the absorbed dose in each of the sensitive silicon volumes, the total energy deposited in each volume was obtained and divided by the total mass of the volume. Each source position was simulated with 10^9^ primary photons for each simulation run. A total of 20 simulation runs were performed for each source position; results from each run were averaged and the standard deviation (*k* = 1) was less than 1%.

Each of the source localization simulations was then repeated with each voxel in the patient geometry overridden to the density of water, to compare the source localization accuracy of the MP with and without the presence of patient‐related inhomogeneities.

To determine the distance of each of the 12 source positions to all detectors in the array (α_i_), a separate group of simulations were first performed to determine the dose deposited in a single detector placed at 10 mm from the source (D_10_), along the z axis (as shown in Fig. 1) in a water phantom. A total of 10 simulations of 10^9^ primary photons were performed for this configuration, and the dose deposited in the single detector averaged across the 10 simulations. This average dose was then used to normalize the dose from each detector in the patient geometry simulations (D_i_), before converting the relative dose to radial distance.

(1) $$\alpha_{i}=\frac{D_{i}}{D_{10}}$$

The radial distance from each detector to each source position can then be determined by converting the relative diode dose to distance via a fit of the TG‐43 parameters calculated in Section 2.C. This approach assumes that the diodes are present within a homogeneous water phantom, when in fact the diode dose was calculated within the heterogeneous patient voxelized phantom. The source positions were determined using an iterative procedure (Appendix 2).^39^, ^40^ Once an initial estimation of the source position is found, a correction factor is then applied to the response of each of the MP detectors to take into account the angular dependence of the detectors. The source position is then re‐estimated using the above method but uses the initial estimated source position of the previous calculation. Finally, the calculated source position is compared to known source positions obtained from the Oncentra Brachytherapy treatment planning system.

## 3 RESULTS

### 3.A TG‐43 simulations

**Figure 4:**
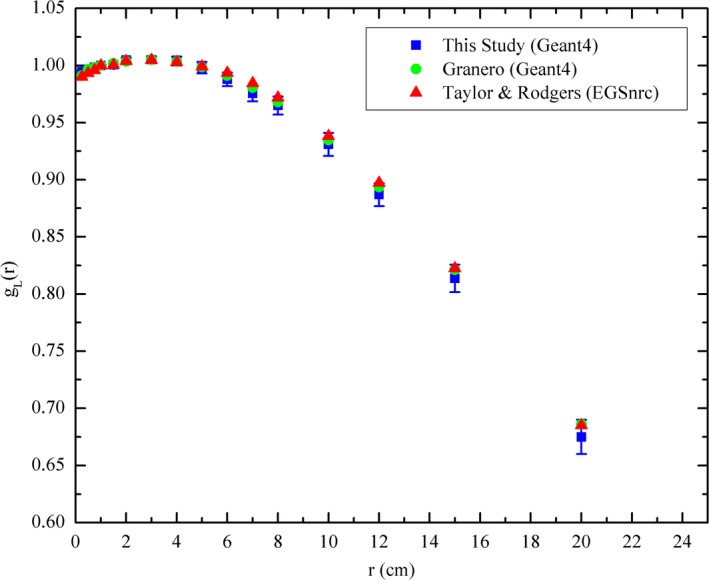
Comparison of calculated radial dose function with studies by Granero^24^ and Taylor & Rogers.^41^

**Figure 5:**
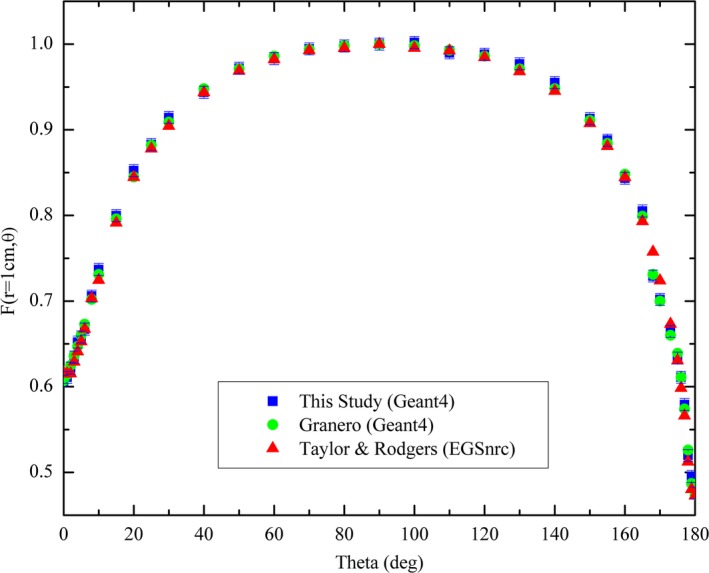
Comparison of calculated 2D anisotropy function at a radial distance of 10 mm with studies by Granero^24^ and Taylor & Rogers.^41^

The radial dose function and anisotropy function (at a radial distance of 10 mm) calculated in this study, using an active length of 3.5 mm for the calculation of G_L_(r,*θ*), are compared to Granero et al.^24^ and Taylor & Rogers^41^ in Figs. 4 and 5, respectively.

The calculated radial dose function from this study was shown to agree (discrepancies < 1%) with both the Granero^24^ and Taylor & Rogers^41^ benchmark datasets within the calculated uncertainty (1.2%) in the radial distance range of 1–200 mm. The 2D anisotropy was also found to agree with the two benchmark datasets to within 1% across the investigated range, verifying the source model and simulation physics were adequate to be used in the study. Larger discrepancies can be observed at polar angles between 0–15° and 165–180° due to the minor variations in source capsule modeling between the studies. In this study, the non‐cable end weld of the stainless steel shell is modeled as a cylinder of length 0.65 mm and diameter 0.85 mm, whereas in the studies by Granero et al.^24^ and Taylor & Rogers^41^ the end weld is modeled as a 0.108 mm thick conical section with a half angle of 23° and the radius of the face being 0.17 mm. This conical section was then attached to a 0.49 mm long solid cylindrical section to complete the end weld. This minor difference in the end weld modeling was not observed to have a significant effect on the calculated anisotropy. There is also a substantial decrease in magnitude of the dose scored in the voxels close to the ends of the source encapsulation due to the significant attenuation through the end welds, this leads to an increased statistical uncertainty and may also contribute to the larger discrepancies in calculated anisotropy between the studies.

### 3.B Source position verification simulations

The average difference between MP predicted and actual source positions was found to be 2.1 ± 0.8 mm (*k* = 1) when all detectors in the array were used in the localization algorithm. Table 1 summarizes the localization results in three dimensions, along with the calculated three‐dimensional difference vector, for different number of detectors used in the source localization algorithm. When not all detectors in the array were used in the source position verification algorithm, the detectors with the highest deposited dose were chosen. As can be seen from Table 1, the MP could localize the source to within 1 mm in the X and Y directions (left/right and superior/inferior directions, respectively). However, it consistently overestimated the distance in the Z direction (anterior/posterior direction), with an average error of 1.9 mm.

**Table 1:** Difference between MP predicted and actual source positions in mm (*k* = 1)

Number of detectors used	9	25	49	81	121
X	0.5 ± 1.0	0.2 ± 0.4	0.0 ± 0.2	0.0 ± 0.2	0.2 ± 0.2
Y	0.5 ± 1.1	0.0 ± 0.5	0.0 ± 0.3	0.2 ± 0.3	0.5 ± 0.5
Z	1.8 ± 0.5	1.8 ± 0.5	1.9 ± 0.5	2.0 ± 0.5	2.0 ± 0.5
3D	2.4 ± 1.0	1.9 ± 0.5	1.9 ± 0.5	2.0 ± 0.5	2.1 ± 0.6

**Figure 6:**
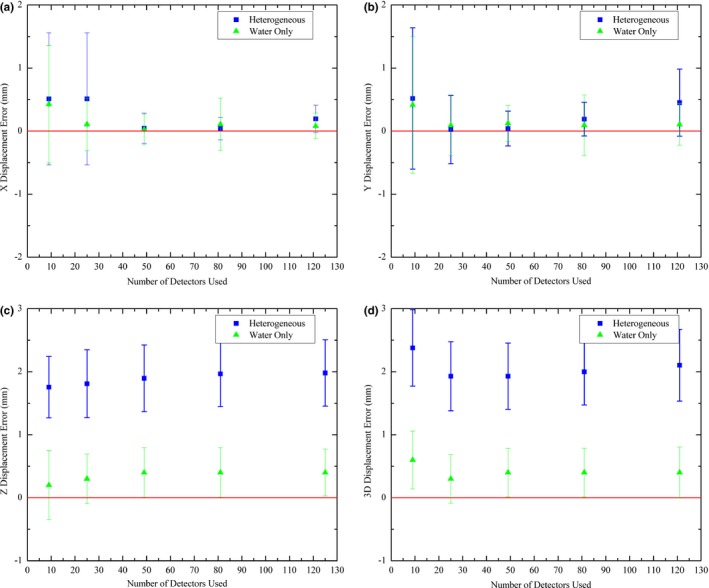
(a) Difference between MP predicted and actual source position for heterogeneous and water only simulations in X direction, (b) difference in Y direction, (c) difference in Z direction, and (d) 3D difference vector. Coordinate system orientation is shown in Figs. 1 and 2.

The source position verification simulations were then repeated using the exact same methods, but with each voxel in the patient geometry assigned a density of water [Figs. 6(a)–6(d)]. The heterogeneous and water only results were compared by means of a Student's *t* test (*P* < 0.05) in each of the X, Y, and Z directions. Only the Z direction differences were found to be statistically significant (*P* < 0.001).

The heterogeneous results were used for a one‐way ANOVA^42^ analysis (*P* < 0.05) to determine if there was a statistically significant difference in the localization accuracy depending on the number of detectors used. The only significant difference was found for the three‐dimensional vector (*P* < 0.001). Subsequent Student's *t* tests were performed to compare the datasets for the three‐dimensional vector. From this, it was found that only the dataset with nine detectors had statistically significant differences to the other groups.

## 4 DISCUSSION

The uncertainties quoted in this study have been evaluated using the combination of both type A and type B uncertainties combined in quadrature, as recommended in AAPM TG43‐U1^27^ and AAPM TG138.^43^ A summary of the uncertainty budget for both the TG‐43 simulations and the source tracking simulations is presented in Table 2.

**Table 2:** Uncertainty analysis for MC simulations used in this study

	Type A	Type B
Statistical variation in absorbed dose determination from repeated MC simulation runs	1%	
Variations of the source geometry from one source to another in manufacturing process^24^		0.5%
Uncertainty in cross‐section library data for Ir‐192^44^		0.5%
Effect of volume averaging on absorbed dose calculation in sensitive volumes^45^		0.1%
Uncertainty in composition of tissues used during source tracking simulations^46^, ^a^		2%
Total TG‐43 simulation uncertainty		1.2%
Total source tracking simulation uncertainty		2.3%

a Applies to source tracking simulations only.

In a water only geometry, the Magic Plate was able to localize the source to within 1 mm. The source localization accuracy, however, was found to decrease with the introduction of inhomogeneities. This decrease in accuracy of source position localization due to the presence of inhomogeneities was found to be primarily in the direction perpendicular to the diode array (z direction). This is due to the source localization algorithm^39^, ^40^ z direction estimate being more sensitive to the changing ratio of primary to secondary photons due to the presence inhomogeneities and increased source to detector distance.

The distance estimate in the z direction is depends directly on the absolute dose deposited in the detector array, whereas source localization in the x and y directions depends only on the relative difference between the distance estimate for each detector and therefore is less sensitive to inhomogeneities. Small inhomogeneities, however, can affect the x and y estimate if a smaller number of detectors are used in the localization algorithm.^39^, ^40^

This indicates that to track the Ir‐192 source with the desired accuracy during HDR prostate brachytherapy treatments a correction may be required, based on density information obtained from the patient CT scan performed prior to treatment. This information could be used, along with a model‐based dose calculation algorithm^37^ built into the localization algorithm to more accurately predict the source to detector distances.

Moreover, it was found that source localization accuracy can be improved with an increased number of detectors used in the localization algorithm. By increasing the number of detectors used in the localization algorithm, a redundancy is built in to reduce the uncertainty introduced due to small heterogeneous media in the patient geometry.^47^ The increased number of detectors is also beneficial due to the relatively isotropic dose profile at large source to detector distances.^40^

Previous studies have shown that source position verification using EPIDs is achievable. However, these studies have also shown that source position verification using EPIDs is restricted by the limited frame rate and readout electronics of the devices and can result in a significant number of dwell positions not being captured by the EPID when performing source position verification.^18^ Source position verification using EPIDs also requires large and expensive systems that have limited availability. This study has shown that similar source position verification accuracy to EPIDs^17^, ^18^, ^19^ may be achieved with the MP system. Furthermore, the MP system has been shown to have a superior timing resolution of less than 1 ms.^20^ As such, the MP delivers a dedicated, inexpensive HDR brachytherapy *in vivo* source position verification system with superior timing resolution that can easily be mass produced and is practical for routine clinical use.

The results of this study, along with previously published experimental results,^16^, ^40^ indicate that the MP will have sufficient sensitivity to detect errors in the order of 1–2 mm during the delivery of HDR prostate brachytherapy treatments when embedded in a carbon fiber couch beneath the patient. Such errors may be due to incorrect catheter connection or incorrect source strength. However, discrepancies less than 1–2 mm in catheter reconstruction, and small movements of catheters in the time between simulation and treatment may not be detectable unless patient‐related heterogeneities can be taken into account during source position verification. Furthermore, one of the most significant challenges for clinical implementation of source position verification using the MP system is the registration of MP and patient coordinate systems. Previous studies have overcome this challenge by using stereoscopic imaging,^17^, ^48^ and electromagnetic tracking technology.^49^, ^50^ Overcoming this challenge will be a focus in future publications.

With the move toward real‐time prostate brachytherapy treatment planning based on transrectal ultrasound imaging, future studies will also aim to examine the effect of the ultrasound probe on source localization accuracy and attempt to optimize the MP position to minimize the effects of heterogeneities.

## 5 CONCLUSION

Source localization using the Magic Plate during a HDR prostate brachytherapy treatment was examined using MC simulations. In a homogenous geometry, the Magic Plate was able to localize the source to within 1 mm. The effect of tissue inhomogeneities in the patient geometry on source localization accuracy was also examined and was found to increase the difference between Magic Plate predicted and known source positions from the brachytherapy treatment planning system to 2.1 ± 0.81 mm (*k* = 1). However, this accuracy can be improved using density information obtained from CT with the MP accurately registered to the patient geometry, making the proposed tool attractive for use as a real‐time *in vivo* QA device in HDR brachytherapy for prostate cancer.

## CONFLICTS OF INTEREST

The authors have no conflicts of interest to report.

## APPENDIX 1 DESCRIPTION OF IR‐192 FLEXISOURCE MODEL

### 1.1

The Ir‐192 core is modeled as a pure Iridium cylinder of length 3.5 mm and diameter 0.6 mm; it has a physical density of 22.42 g/cm^3^.

The active Ir‐192 core is surrounded by a stainless steel shell of length 4.6 mm, 0.85 mm outer diameter, and inner diameter of 0.67 mm. This results in a shell thickness of 0.09 mm. The composition by weight of the stainless steel shell is modeled as follows: Fe 67.92%, Cr 19%, Ni 10%, Mn 2%, Si 1%, and C 0.08%, and the physical density is 7.999 g/cm^3^.

The non‐cable end weld of the stainless steel shell is modeled as a cylinder of length 0.65 mm and diameter 0.85 mm. The cable end weld of the stainless steel shell is modeled as a partial cone of maximum diameter 0.85 mm, minimum diameter of 0.5 mm, and length 0.4 mm.

Finally, the stainless steel cable is modeled as a cylinder of 5 mm length and 0.5 mm diameter, as recommended by Granero et al.^24^ The space between the outer stainless steel shell and the inner Iridium core was modeled as dry air with a physical density of 1.20 mg/cm^3^.

## APPENDIX 2 SOURCE POSITION VERIFICATION ALGORITHM

### 2.1

Based on the derived distances (r_i_) of each diode (i) in the array to the source, as described in Section 2.D, the estimated source position, S_es_(a,b,c) can be calculated. The geometrical distance, d_i_, between S_es_ and the coordinate of the i‐th detector D_i_(x_i_,y_i_,z_i_) is calculated by

(A1) $$d_{i}\left(a,b,c\right)=\sqrt{{\left(a-x_{i}\right)}^{2}+{\left(b-y_{i}\right)}^{2}+{\left(c-z_{i}\right)}^{2}}$$

To determine the true source position, the geometrical distance, d_i_, is fitted to the derived distance, r_i_, by adjusting the estimated source position. Employing a nonlinear least squares fit method to determine the estimated source position. In least squares fitting, the estimate of error assessment can be expressed as the sum of squares of the relative error, Χ^2^

(A2) $$X^{2}\left(a,b,c\right)=\sum_{i=1}^{n}{\left(\frac{d_{i}\left(a_{n},b_{n},c_{n};x_{i},y_{i},z_{i}\right)-r_{i}}{r_{i}}\right)}^{2}$$

and assumes that the derived distance, r_i_, is correct. As there is an uncertainty associated with deriving r_i_, if the estimated source position were equal to the true source position, then calculating the square of the sums of the percentage difference of the value d_i_ and r_i_ would result in a minimum value.

To determine a source position that gives the minimal value to the estimate Χ^2^ a multivariable Newtown's method approach is adopted. The Newton's method is used in this case to determine the roots of a function by finding successively better approximations. In this analysis, it is necessary to determine the minimum values of Χ^2^ for all three dimensions of the estimated source position, and can be expressed as

(A3) $$\frac{\delta X^{2}\left(a,b,c\right)}{\delta a}=\frac{\delta X^{2}\left(a,b,c\right)}{\delta b}=\frac{\delta X^{2}\left(a,b,c\right)}{\delta c}=0$$

The Newton's method for the three source coordinates can be expressed for the *k*‐th iteration as

(A4) $$\begin{array}{cc} a^{k} & =a^{k-1}-\delta a^{k-1}\\ b^{k} & =b^{k-1}-\delta b^{k-1}\\ c^{k} & =c^{k-1}-\delta c^{k-1} \end{array}$$

where δa, δb, and δc are the changes made to the source position to produce the improved approximation. These changes can be determined by solving a set of linear equations, expressed in matrix form as

(A5) $$\left[\begin{array}{ccc} \frac{\delta^{2}X^{2}}{\delta a^{2}} & \frac{\delta^{2}X^{2}}{\delta a\delta b} & \frac{\delta^{2}X^{2}}{\delta a\delta c}\\ \frac{\delta^{2}X^{2}}{\delta b\delta a} & \frac{\delta^{2}X^{2}}{{\delta b}^{2}} & \frac{\delta^{2}X^{2}}{\delta b\delta c}\\ \frac{\delta^{2}X^{2}}{\delta c\delta a} & \frac{\delta^{2}X^{2}}{\delta c\delta b} & \frac{\delta^{2}X^{2}}{\delta c^{2}} \end{array}\right]\left[\begin{array}{c} \delta a\\ \delta b\\ \delta c \end{array}\right]=\left[\begin{array}{c} \frac{\delta X}{\delta a}\\ \frac{\delta X}{\delta b}\\ \frac{\delta X}{\delta c} \end{array}\right]$$

This process is repeated until all δ's are sufficiently small, or until further estimations of the source coordinates fail to reduce Χ^2^. This approach can converge rapidly to a minimum when close, as all three source coordinates are modified in a single iteration.

The initial guess is determined by the coordinates of the detector with the highest response, D_max_(x_max_,y_max_,*z* = 0), as the source is assumed to be closest to this position. The sum of the squares is calculated using the first estimation of the source position,

(A6) $$S_{\mathrm{es}}^{0}=\left(a^{0},b^{0},c^{0}\right)=S_{\mathrm{es}}\left(x_{\max},y_{\max},r_{\max}\right)$$

The source position is then re‐estimated using the above method, but uses the initial estimated source position of the previous calculation.
